# Management of obesity with semaglutide or metformin in patients with antipsychotic-induced weight gain (MOSA): a non-randomised open-label pilot study

**DOI:** 10.1186/s12888-024-06317-7

**Published:** 2024-11-30

**Authors:** Bea Campforts, Marjan Drukker, Therese van Amelsvoort, Maarten Bak

**Affiliations:** 1 Department of Psychiatry & Neuropsychology, Maastricht University, PO Box 616, Maastricht, 6200 MD The Netherlands; 2 Mondriaan Mental Health Centre, Heerlen/ Maastricht, The Netherlands

**Keywords:** Antipsychotic-induced weight gain, GLP-1 agonist, Metformin, Obesity, Semaglutide

## Abstract

**Background:**

Antipsychotic-induced weight gain (AIWG) represents a significant clinical challenge for both patients and clinicians, requiring appropriate interventions to prevent or reverse weight gain in patients using antipsychotics. Glucagon-like peptide 1 (GLP-1) agonists represent a novel approach to the management of obesity that has recently attracted considerable attention. Semaglutide (a GLP-1 agonist) has been demonstrated to result in notable weight loss. The present study investigates whether semaglutide is equally effective in achieving weight loss in patients with AIWG.

**Methods:**

A prospective, non-randomised cohort study was conducted with the objective of evaluating the efficacy and safety of oral semaglutide for the treatment of AIWG in routine outpatient clinical practice. Subsequently, the results were compared with those of a control group of AIWG patients taking metformin.

**Results:**

After 16 weeks, the mean body weight loss was 4.5 kg (95% confidence interval (CI), -6.7 to -2.3 kg; *p* < 0.001) in the semaglutide group (*n* = 10) versus 2.9 kg (95% CI, -4.5 to -1.4 kg; *p* < 0.001) in the metformin group (*n* = 26). This corresponds to an average body weight loss of 4% for semaglutide, and 2.5% for metformin. The respective reductions in body mass index (BMI) and waist circumference were -1.7 kg/m2 (95% CI, -2.4 to -1.0 kg/m2; *p* < 0.001) and -6.8 cm (95% CI, -9.7 to -3.8 cm; *p* < 0.001) for semaglutide. The observed reductions for metformin were -0.8 kg/m2 (95% CI, -1.4 to -0.3 kg/m2; *p* = 0.001) and -3.4 cm (95% CI, -5.4 to -1.3 cm; *p* = 0.001). The differences between the two groups were not statistically significant. In both groups, adverse effects were typically mild and transient, predominantly nausea. Furthermore, psychiatric symptoms were reduced, and quality of life improved.

**Conclusions:**

Oral semaglutide represents a viable, effective, and safe treatment option for psychiatric patients. However, further investigation is required to corroborate these findings.

## Introduction

Antipsychotic treatment constitutes a fundamental component of the management of psychotic disorders [1, 2]. A recent network meta-analysis (NMA) has demonstrated that the majority of antipsychotics (APs) are effective in treating psychiatric symptoms associated with schizophrenia [3]. However, the most effective APs carry a higher risk of weight gain [4, 5], which can result in severe general adverse health effects [6]. Consequently, antipsychotic-induced weight gain (AIWG) represents a significant clinical challenge for both patients and clinicians.

Guidelines emphasise the importance of monitoring the adverse effects of APs [1, 7–10] and recommend initiating treatment with APs that cause the least weight gain and monitoring weight gain. Despite these precautions, a considerable number of patients will still develop AIWG and require appropriate interventions. Previous research indicates that lifestyle counselling, exercise interventions, antipsychotic substitution, discontinuation and dose reduction may represent potential avenues for treatment, although their efficacy is limited [11–15].

Pharmacotherapy represents a relatively new approach to the management of AIWG and obesity [16]. Currently, a number of pharmacological options are available for the treatment of AIWG, with metformin and topiramate being the most extensively researched [17, 18]. Prior research has demonstrated the feasibility, effectiveness and safety of metformin as a pharmacological intervention for AIWG [18]. A systematic review and meta-analysis indicated that the addition of metformin resulted in a modest weight loss of −3.3 kg over a period of 12–24 weeks [17]. Metformin has been approved for the treatment of AIWG, with established guidelines for its use [19].

Another group of drugs are glucagon-like peptide-1 (GLP-1) antidiabetics, such as liraglutide and semaglutide. These antidiabetics have been demonstrated to result in significant weight loss in non-diabetic obese patients, with semaglutide being among the most efficacious [20, 21]. The side-effect profile is relatively mild, with the most common adverse event being nausea and vomiting [20].

To date, there is limited evidence regarding the use of GLP-1 agonists in psychiatric patients for the treatment of AIWG. A recent meta-analysis found that exenatide and liraglutide resulted in a mean weight loss of −2.5 kg and −4.7 kg, respectively, over a period of 12–24 weeks [22]. The use of semaglutide in patients with AIWG is considered promising given its potential benefits for obese patients with a range of physical problems that are associated with obesity [23]. Furthermore, the administration of a GLP-1 agonist and the associated weight loss may have a positive impact on physical function, psychological well-being and quality of life [20, 23, 24]. However, apart from a recently published number of cases [25], no studies have yet been conducted on the use of semaglutide in patients with AIWG.

Semaglutide has recently become available for oral prescription. Recent studies have demonstrated that once-daily oral semaglutide is at least as effective as subcutaneous injectable semaglutide for weight loss in patients with [26] and without type 2 diabetes mellitus (DM2) [27]. Furthermore, the adverse effects of oral and subcutaneous semaglutide are comparable [27], offering a valuable alternative for patients and physicians who prefer oral medication as a more convenient route of administration in a primary care setting [27].

The principal objective of the MOSA study was, therefore, to assess the feasibility of administering oral semaglutide to patients with AIWG in routine outpatient clinical practice. To this end, a prospective, non-randomised cohort study was conducted with the objective of assessing the efficacy of semaglutide in terms of weight loss and tolerability for the treatment of AIWG, in comparison with the active control condition of metformin. Furthermore, any adverse effects that may have manifested were monitored and recorded. In addition, the incidence and severity of psychiatric symptoms, as well as quality of life (QoL), were evaluated in both groups.

## Methods

### Study design

This prospective, non-randomised, open-label cohort study with two active medication arms (semaglutide and metformin), was conducted between September 2021 (first patient, first visit) and March 2024 (last patient, last visit) at Mondriaan, a mental health care institution in the Netherlands. The study was approved by the Mondriaan Mental Health Care Institutional Review Board (IRB no. CWO21017) and conducted in accordance with the Declaration of Helsinki [28] and Good Clinical Practice. Prior to their participation in the study, patients were informed verbally and in writing and gave written informed consent. The treating clinicians were asked to prescribe either metformin or semaglutide, and they were similarly informed and required to consent to participate with their patient.

### Study participants

The study included patients aged 16 years and over who were receiving psychiatric care and were taking AP medication. Patients were eligible for inclusion in the study if they had a body mass index (BMI) of 27.5 kg/m^3^ or more and stable psychopathology, defined as psychiatric symptoms under control and no anticipated change in psychiatric medication during the study period. Furthermore, it was imperative that psychiatric medications and doses remained unchanged throughout the study period, and that no additional psychiatric medications (except for the study medication) were introduced during this period. Patients were excluded if they had known type 1 or type 2 diabetes mellitus, diabetic retinopathy, thyroid tumours or a family history of thyroid tumours, somatic comorbidity affecting weight, or known complications of metformin or semaglutide. Furthermore, patients were excluded if they exhibited neurocognitive impairment, were using amphetamines or cocaine-related substances, were pregnant or lactating, or were of childbearing age and not using contraception. The use of nicotine or cannabis was common in this population and was allowed, provided it remained stable throughout the course of the study.

### Study procedures and allocation to treatment

This prospective cohort study was conducted to assess the efficacy and safety of weight-loss medications for the treatment of AIWG in routine outpatient clinical practice. Patients were invited to participate in the study on an entirely voluntary basis and were at liberty to stop taking the weight-loss medication or withdraw from the study at any time.

The decision to utilise a pharmacological intervention to treat AIWG was made by the clinician and the patient as part of regular clinical care. The choice between semaglutide and metformin was based on the patient's needs and preferences. Both the patient and the clinician were provided with written and verbal information about the clinical aspects of semaglutide and metformin. This included details of the respective mechanisms of action, potential benefits and risks, and any relevant contraindications. This was done to ensure that the patient and clinician could make an informed decision based on a comprehensive understanding of the available information.

The prescription of semaglutide was done in two steps in accordance with the guidelines. For the initial 30 days, the patient started with one 3 mg tablet of semaglutide once a day [29]. If there were no contraindicating side effects, the dose was increased to 7 mg once daily. This dose was maintained until week 16. For safety reasons and because semaglutide had not previously been studied in this population of psychiatric patients, it was decided not to increase the dose beyond 7 mg. Participants were instructed to take the tablet in the morning on an empty stomach with up to half a glass of water (equivalent to 120 ml) and to wait at least 30 min before eating, drinking or taking any other oral medication. Waiting less than 30 min will reduce absorption. Tablets should not be split, crushed or chewed as it is not known whether this affects the absorption of semaglutide [29]. The dose of metformin should be titrated according to standard prescribing guidelines with a range of 500–2000 mg/day. In this study, the dose was initiated at 500 mg once daily for 30 days and then increased to 500 mg twice daily in the absence of contraindications.

All patients enrolled in the study received standard clinical care and were advised on lifestyle changes, such as dietary changes and exercise, in accordance with standard clinical practice. Furthermore, the treating clinicians were encouraged to address lifestyle issues with their patients, as a matter of general good practice. However, no specific lifestyle intervention was introduced in addition to the study intervention of semaglutide or metformin. All physical examinations and psychometric assessments were conducted by the researcher, who was not involved in the treatment of the patient, but was aware of the study medication prescribed, either metformin or semaglutide.

**Fig. 1:**
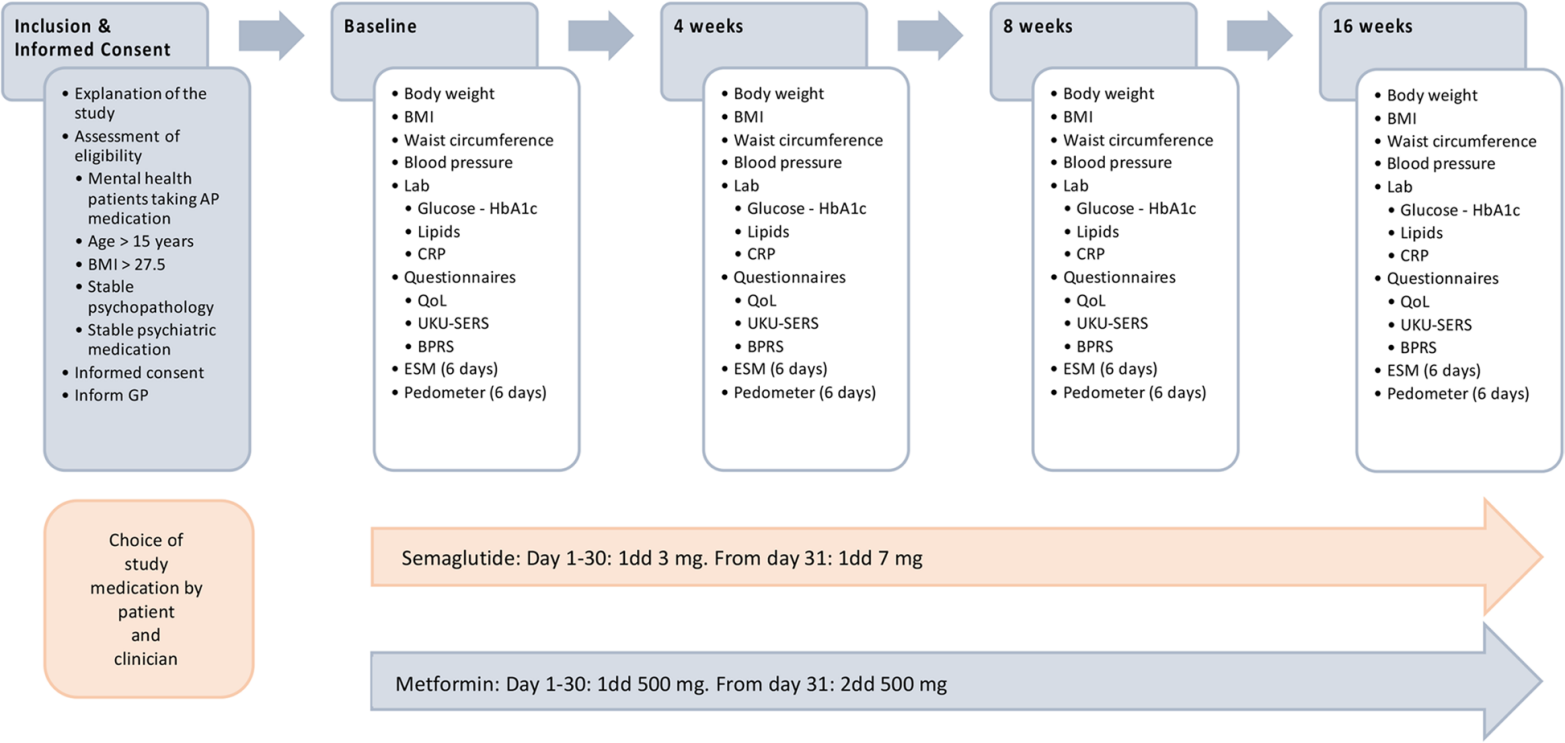
Timeline of the MOSA study. AP, antipsychotic; BMI, body mass index; GP, general practitioner; HbA1c, glycated haemoglobin; CRP, C-reactive protein; QoL, quality of life; UKU-SERS, UKU side effect rating scale; BPRS, brief psychiatric rating scale; ESM, experience sampling method

The study was conducted in two cohorts, one receiving metformin and the other receiving semaglutide. Both cohorts were followed for a period of 16 weeks. Assessments were conducted at baseline and at 4 weeks, 8 weeks and 16 weeks (see Fig. 1 for an overview of the study).

### Adverse events

As this was an open-label study, the prescribing clinician was aware of the specific medication being administered. In the event of a serious adverse reaction that the patient and/or treating clinician believed to be caused by or related to the administration of semaglutide or metformin, the medication was discontinued, and the adverse event was reported to the investigator. Furthermore, the study team informed the general practitioner (GP) of the prescribed medication, either metformin or semaglutide, and explained the purpose of the study. In the event of adverse effects, the GP would be able to ensure that the adverse effects were adequately addressed.

### Outcomes and measures

The primary outcomes were changes in body weight (kg), BMI (kg/m^2^) and waist circumference from baseline to week 16. To ensure consistency and reliability, body weight was measured using the same digital scale at each assessment. Waist circumference was measured at the level of the navel, midway between the lowest rib and the top of the hipbone. Secondary outcomes included blood pressure measured while seated for five minutes and changes in fasting plasma glucose, glycated haemoglobin (HbA1c), lipids (high-density lipoproteins (HDL), low-density lipoproteins (LDL), triglycerides and total cholesterol) and C-reactive protein levels. To assess changes in physical complaints and side effects, we used the self-report version of the UKU Side Effect Rating Scale (UKU-SERS), a comprehensive side effect rating scale developed for use in clinical drug trials and routine clinical practice [30]. The UKU scale comprises 48 items, each of which is scored on a 4-point scale (0–1–2–3). Adverse events were documented based on patients' feedback on each of the 48 items listed on the UKU scale. Furthermore, changes in general psychopathology were assessed using the Brief Psychiatric Rating Scale (BPRS) [31, 32]. The 18-item scale is a standard instrument used in clinical care at Mondriaan to assess levels of psychotic symptoms, mood symptoms and functioning. The BPRS was administered by a trained physician or psychologist. To assess changes in quality of life, the self-report Kemp Quality of Life Scale (KQOL) [33] was employed. This is a Likert scale, ranging from 1 to 7, with 1 indicating a very distressing quality of life and 7 indicating a high quality of life.

### Statistical analysis

All statistical analyses were conducted using Stata for Mac version 16.1 [34]. Continuous variables were described as means and standard deviations, while categorical variables were presented as numbers and percentages. The significance of between-group differences in categorical variables was assessed using a Fisher's exact test, as the cell numbers were too low for a chi-square test. Continuous variables were analysed using independent samples t-tests or Mann–Whitney tests, depending on the normality of the data distribution.

All efficacy analyses were performed using a modified intention-to-treat principle; all participants who received at least one dose of the study medication (semaglutide or metformin) and had at least one post-baseline assessment, were included in the efficacy analyses. All participants who received at least one dose of the study medication (semaglutide or metformin) were included in the safety analyses. Mixed effect linear regression is ideally suited to analyse using the modified intention-to-treat principle because it included multiple time points per person and all available data are modelled and extrapolated to predict missing data [35]. Thus, a mixed-effects linear regression model analysis was employed to assess the effect of semaglutide and metformin on continuous outcomes, including body weight, BMI, waist circumference, and secondary outcome variables over time. The model included the baseline value of the relevant variable together with the covariates age, sex, baseline BPRS score and baseline QoL score. In addition, post hoc sensitivity analyses were conducted to assess the robustness of the primary analyses. To assess changes over time in side effects as rated by the UKU scale, difference scores for side effects were calculated by subtracting the baseline score on the items of the UKU side effects rating scale from the actual scores over time. Subsequently, a mixed-effects linear regression model was fitted, incorporating the aforementioned covariates: age, sex, baseline BPRS score, and baseline QoL score. All statistical tests were two-tailed with statistical significance set at 0.05.

## Results

### Participants and baseline characteristics

**Fig. 2:**
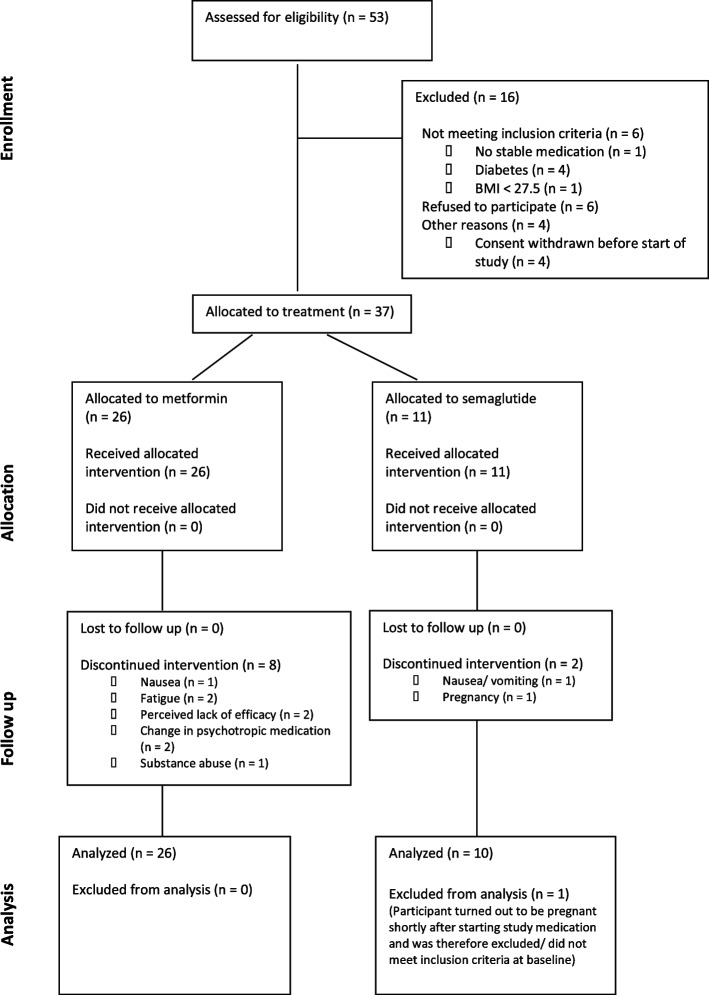
Study diagram of the patient flow according to CONSORT 2010 statement. All participants who received at least one dose of the study medication (semaglutide or metformin) and had at least one post-baseline assessment were included in the efficacy analyses. Participants who did not meet the inclusion criteria at baseline or who discontinued the intervention before the first visit at week 4 were excluded. One participant in the semaglutide group did not meet the inclusion criteria at baseline and was excluded from the analyses. Eight participants in the metformin group and one participant in the semaglutide group discontinued or were excluded after the first post-baseline visit and were therefore included in the efficacy analyses without completing 16 weeks of treatment. All participants who received at least one dose of semaglutide or metformin were included in the safety analyses. BMI is body mass index (calculated as weight in kilograms divided by height in metres squared)

A total of 53 patients were referred and assessed for eligibility between August/September 2021 and October 2023. A total of 16 patients were excluded from the study, 6 because they did not meet the inclusion criteria, 6 refused to participate and 4 withdrew their consent before the start of the study (Fig. 2). A total of 37 patients were enrolled and allocated to treatment with semaglutide (*n* = 11) or metformin (*n* = 26), and 10 patients dropped out, giving a drop-out rate of 27%. Two patients discontinued treatment in the semaglutide group: one due to severe nausea and vomiting and one because she discovered that she was pregnant shortly after initiating the study medication, despite using contraception. As semaglutide is contraindicated in pregnancy, treatment was discontinued, and the patient was excluded from further analysis since she did not meet the inclusion criteria at baseline. Eight patients in the metformin group discontinued the study prematurely: 1 patient due to dyspepsia, 2 due to fatigue, 2 due to a lack of perceived efficacy, 2 due to worsening psychiatric symptoms and a consequent change of psychotropic medication, and 1 due to substance abuse. A total of 27 patients (9 in the semaglutide group, 18 in the metformin group) completed the study. A total of 36 patients who received at least one dose of study medication (10 in the semaglutide group, 26 in the metformin group) and had at least one post-baseline assessment, were included in the efficacy analyses (Fig. 2). All participants who received at least one dose of semaglutide or metformin were included in the safety analyses (*n* = 37).

At baseline, there were no statistically significant differences between the groups with respect to age, sex, diagnosis, psychiatric symptoms, weight, height, BMI, waist circumference, fasting plasma glucose, lipids and C-reactive protein, except for HbA1c (Table 1). Patients in both groups were treated with different antipsychotics (both monotherapy and polypharmacy), including first-generation antipsychotics (bromperidol, flupentixol, haloperidol, penfluridol and pimozide) and second-generation antipsychotics (amisulpride, aripiprazole, clozapine, olanzapine, paliperidone, quetiapine and risperidone), with no differences between groups. The use of antidepressants and mood stabilisers did not differ between groups. (Table 1).

**Table 1:** Baseline Characteristics

Characteristic	Metformin (*n* = 26)	Semaglutide (*n* = 10)	*p*-value
**Sociodemographic**			
Age (y), mean (SD)	42.8 (11.2)	38.3 (7.3)	0.24^t^
Gender N (%)			0.27^f^
Female	15 (57.7%)	8 (80%)	
Male	11 (42.3%)	2 (20%)	
**Diagnosis N (%)**			0.76^f^
Schizophrenia	5 (19.2%)	2 (20%)	
Schizophrenia spectrum disorder	8 (30.8%)	2 (20%)	
Schizoaffective disorder	2 (7.7%)	1 (10%)	
Delusional disorder	1 (3.8%)	0 (0%)	
Bipolar disorder	3 (11.6%)	3 (30%)	
Major depressive disorder	1 (3.8%)	1 (10%)	
Personality disorder	6 (23.1%)	1 (10%)	
**Clinical characteristics, mean (SD)**			
Body weight (kg)	113.4 (23.9)	116.3 (24.0)	0.74^t^
Height (cm)	173.8 (13.1)	170.3 (8.6)	0.44^t^
BMI (kg/m2)	37.5 (6.2)	40.1 (7.5)	0.29^t^
Waist circumference (cm)	121.3 (17.3)	121.1 (19.0)	0.97^t^
Systolic blood pressure (mmHg)	121.5 (19.8)	123.7 (16.8)	0.76^t^
Diastolic blood pressure (mmHg)	83.3 (11.1)	86.3 (10.7)	0.45^t^
**Blood biochemistry, mean (SD)**^**a**^			
HbA1c (mmol/mol)	39.2 (5.5)	32.6 (3.0)	0.004^t^
HbA1c (%)	5.8 (0.5)	5.1 (0.3)	0.004^t^
Fasting plasma glucose (mmol/L)^b^	6.4 (1.5)	5.3 (0.5)	0.07^U^
Fasting lipids (mmol/L)			
Total cholesterol	5.3 (1.1)	4.9 (0.8)	0.50^t^
HDL	1.1 (0.3)	1.1 (0.2)	0.81^t^
LDL	3.3 (0.9)	3.0 (0.8)	0.45^t^
Triglycerides	2.3 (1.6)	2.0 (0.7)	0.91^U^
C-reactive protein	11.8 (10.4)	9.1 (11.0)	0.29^U^
**Rating scales, mean (SD) score**			
Brief Psychiatric rating scale^c^	33.6 (8.1)	34.6 (3.8)	0.77^t^
Quality of Life^d^	4.6 (1.6)	4.0 (1.5)	0.25^t^
**Treatment, N (%)**			
** Antipsychotics**			0.60^f^
Amisulpride	2 (7.7%)		
Aripiprazole	6 (23.1%)	1 (10%)	
Bromperidol		1 (10%)	
Clozapine	3 (11.6%)	3 (30%)	
Clozapine and aripiprazole		1 (10%)	
Clozapine and quetiapine	1 (3.8%)		
Flupentixol	1 (3.8%)		
Haloperidol		1 (10%)	
Olanzapine	4 (15.4%)	2 (20%)	
Olanzapine (IM)	1 (3.8%)		
Paliperidone (IM)	2 (7.7%)		
Penfluridol	1 (3.8%)		
Pimozide	1 (3.8%)		
Quetiapine	3 (11.6%)	1 (10%)	
Risperidone	1 (3.8%)		
** Antidepressants**			0.85^f^
Amitriptyline	1 (3.8%)		
Bupropion	1 (3.8%)		
Citalopram	2 (7.7%)	1 (10%)	
Fluoxetine	1 (3.8%)	1 (10%)	
Fluvoxamine	1 (3.8%)		
Mirtazapine	1 (3.8%)		
Nortriptyline	1 (3.8%)		
Sertraline		2 (20%)	
Venlafaxine		1 (10%)	
** Mood stabilizers/ anticonvulsants**			1.0^f^
Lithium		1 (10%)	
Valproic acid	1 (3.8%)	1 (10%)	
Topiramate	1 (3.8%)	1 (10%)	

Data are mean (SD); range or percentage

*Abbreviations*: *BMI* Body mass index, *HbA1c* glycated haemoglobin *HDL* High-density lipoprotein, *IM* Intramuscular, *LDL* Low-density lipoprotein, *SD* Standard deviation

^t^Two independent sample t test with equal variance

^f^fishers exact test

^U^ Mann–Whitney test for non-normal distributed values

^a^Data are missing from five participants in the metformin arm and one in the semaglutide arm

^b^Four patients on metformin, 2 on semaglutide were not sober at blood collection

^c^Scores range from 18 to 126, with higher scores indicating poorer mental health

^d^Scores range from 1 to 7, with higher scores indicating better quality of life

### Body weight and metabolic variables

After 16 weeks of treatment, the mean weight loss observed in the semaglutide group was −4.5 kg (95% CI, −6.7 to −2.3 kg; *p* < 0.001), compared to a mean loss of with −2.9 kg (95% CI, −4.5 to −1.4 kg; *p* < 0.001) in the metformin group. The mean reduction in BMI was −1.7 (95% CI, −2.4 to −1.0; *p* < 0.001) for semaglutide and −0.8 (95% CI, −1.4 to −0.3; *p* = 0.001) for metformin. The mean reduction in waist circumference was −6.8 cm (95% CI, −9.7 to −3.8 cm; *p* < 0.001) for semaglutide and −3.4 cm (95% CI, −5.4 to −1.3; *p* = 0.001) for metformin, respectively. The mean differences between semaglutide and metformin were found to be borderline statistically significant for both BMI (−0.9; 95% CI, −1.7 to 0.0; *p* = 0.06) and waist circumference (−3.4 cm; 95% CI, −7.0 to 0.2 cm; *p* = 0.06) (Table 2). The results of sensitivity analyses in which outliers were excluded from the data set demonstrated that the observed changes in weight (*p* = 0.001), BMI (*p* = 0.002) and waist circumference (*p* = 0.001) remained statistically significant for both groups. However, no statistically significant mean differences were observed between semaglutide and metformin for these variables.

**Table 2:** Change in End Points From Baseline to Week 16

Characteristic	Semaglutide (*n* = 10)	*p*-value	Metformin (*n* = 26)	*p*-value	Covariate Adjusted Estimated Difference, Semaglutide vs Metformin (95% CI) at 16 wks^a^	*p*-value
**Clinical characteristics, mean (SE)**						
Body weight (kg)	**−4.5 (1.1)**	** < 0.001**	**−2.9 (0.8)**	** < 0.001**	−1.6 [−4.3 -to 1.1]	0.24
BMI (kg/m2)	**−1.7 (0.4)**	** < 0.001**	**−0.8 (0.3)**	**0.001**	−0.9 [−1.7 to −0.0]	0.06
Waist circumference (cm)	**−6.8 (1.5)**	** < 0.001**	**−3.4 (1.0)**	**0.001**	−3.4 [−7.0 to 0.2]	0.06
Systolic blood pressure (mmHg)	−9.7 (5.0)	0.05	**−7.7 (3.2)**	**0.02**	−2.0 [−13.5 to 9.6]	0.74
Diastolic blood pressure (mmHg)	−1.2 (2.8)	0.66	−1.0 (1.8)	0.56	−0.2 [−6.6 to 6.2]	0.98
**Blood biochemistry, mean (SE)**^**b**^						
HbA1c (mmol/mol)	−1.8 (1.2)	0.12	−0.2 (0.9)	0.84	−1.6 [−4.5 to 1.2]	0.25
HbA1c (%)	−0.2 (0.1)	0.11	−0.0 (0.1)	0.84	−0.2 [−0.4 to 0.1]	0.25
Fasting plasma glucose (mmol/L)	−0.4 (0.4)	0.43	−0.2 (0.3)	0.48	−0.1 [−1.2 to 1.0]	0.82
Fasting lipids (mmol/L)						
Total cholesterol	+ 0.1 (0.3)	0.68	−0.0 (0.2)	0.84	+ 0.2 [−0.5 to 0.8]	0.65
HDL	−0.0 (0.0)	0.63	+ 0.0 (0.0)	0.96	−0.0 [−0.1 to 0.1]	0.68
LDL	+ 0.2 (0.2)	0.45	−0.2 (0.2)	0.32	+ 0.3 [−0.2 to 0.9]	0.23
Triglycerides	+ 0.2 (0.3)	0.60	+ 0.2 (0.2)	0.38	−0.0 [−0.8 to 0.7]	0.93
C-reactive proteine (mg/L)	−4.1 (2.8)	0.15	−3.4 (2.2)	0.11	−0.6 [−7.6 to 6.3]	0.85
**Rating scales, mean (SE) score**						
Brief Psychiatric rating scale^c^	**−3.3 (1.3)**	**0.01**	**−2.1 (0.9)**	**0.03**	−1.3 [−4.4 to 1.9]	0.44
Quality of Life^d^	** + 1.4 (0.4)**	** < 0.001**	+ 0.3 (0.2)	0.18	**+ 1.1 [0.2 to 1.9]**	**0.01**

*Abbreviations: BMI* Body mass index, *CI* Confidence interval, *HbA1c* glycated haemoglobin, *HDL* High-density lipoprotein, *LDL* Low-density lipoprotein, *SE* Standard error

^a^Estimated treatment differences between groups were calculated using a mixed-model analysis of covariance. The model includes the baseline value of the relevant variable together with the covariates age; sex; and baseline BPRS-score

^b^Data are missing from five participants in the metformin arm and one in the semaglutide arm

^c^Scores range from 18 to 126, with higher scores indicating poorer mental health

^d^Scores range from 1 to 7, with higher scores indicating better quality of life

Significant differences are shown in bold

Furthermore, metformin was found to reduce systolic blood pressure by 7.4 mmHg (95% CI, −13.6 to −1.2 mmHg; *p* = 0.02), although this difference was not statistically significant in comparison to semaglutide (−9.7 mmHg; 95% CI, −19.5 to 0.1 mmHg; *p* = 0.05) (Table 2).

No significant changes were observed in fasting plasma glucose, glycated haemoglobin/HbA1c, C-reactive protein and fasting measures of total cholesterol, HDL cholesterol, LDL cholesterol and triglycerides (Table 2).

### Adverse events

The most frequently observed adverse events were gastrointestinal in nature and predominantly occurred during the initiation at the start of treatment or during dose escalation (Table 3). The results of the longitudinal analyses indicated that there were no statistically significant differences between the semaglutide and metformin groups with regard to the incidence of nausea and vomiting. A statistically significant increase of nausea incidence was observed in both groups at week 4 (*p* = 0.01), with a subsequent decline. The incidence of diarrhoea was significantly higher in the metformin group at weeks 4 (*p* = 0.003) and 8 (*p* = 0.01), but no longer at week 16. No significant temporal changes were observed in the incidence of constipation. The majority of adverse effects were mild, and only two patients discontinued treatment due to gastrointestinal side effects. One patient in the semaglutide group discontinued treatment due to severe nausea and vomiting, while the other patient in the metformin group discontinued treatment due to nausea and dyspepsia. Two patients in the metformin group who discontinued treatment reported fatigue and generalised weakness. The longitudinal analysis indicated that, although fatigue was similarly prevalent in both groups at baseline, it exhibited a gradual decline over time, reaching a significant decrease at week 16 (*p* = 0.02). Two patients exhibited a worsening of psychiatric symptoms during metformin treatment. One patient was hospitalised for dehydration, which was not considered to be related to metformin treatment (Table 3). The attrition rate due to adverse effects was 9.1% in the semaglutide group (*n* = 1) and 23.1% in the metformin group (*n* = 6). No statistically significant between-group difference was observed (*p* = 0.65).

**Table 3:** Adverse Events/ Reactions and Serious Adverse Events^a^

Adverse event or reaction	Semaglutide (*n* = 11)	Metformine (*n* = 26)	*p*-value^b^
**Gastrointestinal tract**			
Nausea	4/11 (36.4)	2/26 (7.7)	0.05
Vomiting	1/11 (9.1)	2/26 (7.7)	1.00
Diarrhoea	0 (0.0)	6/26 (23.1)	0.15
Constipation	2/11 (18.2)	4/26 (15.4)	1.00
**Nervous system**			
Fatigue	6/11 (54.5)	13/26 (50.0)	1.00
Headache	2/11 (18.2)	6/26 (23.1)	1.00
**Cardiovascular**			
Palpitations/ tachycardia	0 (0.0)	2/26 (7.7)	1.00
Dizziness (orthostatic)	1/11 (9.1)	4/26 (15.4)	1.00
**Psychiatric**			
Worsening of symptoms	0 (0.0)	2/26 (7.7)	1.00
**Serious Adverse events**			
Total No	0	1	1.00
**Admission to hospital for somatic reasons**	0	1	1.00
**Admission to hospital for psychiatric reasons**	0	0	

Data are number of participants (%)

^a^All participants who received at least 1 dose of semaglutide or metformin were included in the safety analyses

^b^Fisher's exact test *p*-value

### Psychiatric symptoms and quality of life

At 16 weeks, a small but significant reduction in psychiatric symptoms was observed, as evidenced by a mean reduction of −3.3 points on the BPRS (95% CI, −5.9 to −0.7; *p* = 0.01) for semaglutide and −2.1 points (95% CI, −3.9 to −0.3; *p* = 0.03) for metformin. No statistically significant differences were identified between the two groups.

Furthermore, there was a significant improvement in quality of life in the semaglutide group (+ 1.4; 95% CI, 0.7 to 2.1; *p* < 0.001), but not in the metformin group (+ 0.3; 95% CI, −0.1 to 0.8; *p* = 0.18). The mean difference between semaglutide and metformin was statistically significant, with an estimated mean difference of + 1.1 (95% CI, 0.2 to 1.9; *p* = 0.01) (Table 2).

## Discussion

This prospective study was the first to evaluate the effects of oral semaglutide treatment in obese people receiving antipsychotic medication. After 16 weeks of treatment with semaglutide, significant reductions in weight, body mass index (BMI) and waist circumference were observed. In general, various outcomes showed that weight loss was larger in the semaglutide group than in the metformine group, but differences were not statistically significant.

### Weight loss

By the end of the intervention period, the mean body weight loss was 4.5 kg in the semaglutide group and 2.9 kg in the metformin group. This accounted for an average of 4% body weight loss over 4 months for semaglutide, and 2.5% for metformin. The corresponding reductions in BMI were −1.7 and −0.8 for semaglutide and metformin, respectively. The reduction in waist circumference observed in both groups was 6.8 cm and 3.4 cm, respectively.

These findings are consistent with those of a recent retrospective chart review investigating the addition of subcutaneous semaglutide for AIWG [25]. The mean weight loss observed in this study was 4.6 kg over a three-month period and 5.2 kg over a six-month period. However, the majority of patients in this study were concomitantly administered metformin, and the dosage of semaglutide employed was higher (on average 0.7 mg/week subcutaneously) than that employed in this study (7 mg per day orally, corresponding to a weekly dose of 0.25 mg subcutaneously). The findings are also consistent with those achieved with subcutaneous liraglutide, another GLP-1 agonist, for the treatment of AIWG. A recent meta-analysis indicated a mean weight loss of 4.7 kg with liraglutide over a period of 3 to 6 months, corresponding to a reduction in BMI of −1.5 BMI points [22]. These findings were derived from the results of two randomised controlled trials (RCTs) [36, 37] and one cohort study [38] that examined the use of liraglutide in this population. A similar conclusion can be drawn regarding the results achieved with the use of metformin in this patient population. A previous meta-analysis indicated a mean weight loss of 3.3 kg over a period of 3 to 6 months [17].

### Safety

The safety and tolerability of semaglutide and metformin were also evaluated in this population. The most common adverse events were gastrointestinal in nature, mainly nausea, and occurred most frequently at the start of treatment or during dose escalation. However, the majority of adverse events were mild and only two patients discontinued treatment due to gastrointestinal side effects. These findings are consistent with those of previous studies investigating semaglutide [25] or other GLP-1 agonists [36–38] in this population.

### Psychiatric symptoms and quality of life

Furthermore, treatment with semaglutide or metformin did not adversely affect psychiatric health, which is consistent with the findings of previous studies [36, 38]. In fact, a small but significant improvement in psychiatric symptoms was observed at the end of the 16-week intervention period for both treatment groups. The assessment of changes in psychopathology represents a relevant area of investigation, particularly in light of the interaction between GLP-1 and the cholinergic and dopamine-derived motivational system [39] and the established association between obesity and psychopathology [40]. A previous meta-analysis indicated that GLP-1 agonists are not associated with an increase in psychopathology as an adverse effect [22]. The potential beneficial effect of GLP-1 agonists on psychiatric symptoms has not been consistently documented in the literature. However, previous research has indicated that GLP-1 agonists may exert a beneficial effect on depressive symptoms and quality of life [23, 24]. The present study observed a significant improvement in self-perceived quality of life in patients using semaglutide, which was not consistently evaluated or observed in previous studies [25, 36]. This topic merits further investigation.

### Individual variations

Although the mean weight loss for semaglutide and metformin was 4% and 2.5%, respectively, there were notable individual variations. Some patients exhibited a weight loss of up to 17% for semaglutide and 13% for metformin, whereas others did not lose any weight and even exhibited slight weight gain. Following the exclusion of outliers from the data set in sensitivity analyses, the observed changes in weight, body mass index (BMI) and waist circumference remained statistically significant. In order to ascertain the underlying causes of these discrepancies, the MOSA study included an experience sampling method (ESM) app and a pedometer as research tools. The findings from this ESM and pedometer data will be presented in a subsequent article. The ESM data allow for the the investigation of a number of underlying factors, including dietary patterns, activity levels, exercise routines, mood, and other ecological variables, that may explain these differences [41].

### Future research

Further research is required to determine the optimal duration of semaglutide and other GLP-1 agonist therapies and to identify effective strategies for maintaining and discontinuing the weight-reducing GLP-1 agonist treatment. Prior research has indicated that patients on average regained two-thirds of the weight lost following the cessation of semaglutide [42]. Currently, several options are being investigated, including slow tapering strategies and low-dose continuation of semaglutide [43, 44]. In accordance with the guidelines, the addition of weight loss medications is preferable to be combined with lifestyle interventions [45]. This approach may enhance the efficacy of weight loss medications and reduce the risk of weight regain following cessation of weight loss medications [43]. It is well established that lifestyle interventions exert an influence on both weight gain and psychiatric symptomatology [45]. However, patients with severe mental illness (SMI) frequently demonstrate decreased motivation and an inability to adapt their diet and activity patterns [46, 47], which limits the effectiveness of lifestyle interventions [12]. It is hypothesised that initiating a pharmacological intervention may be beneficial in overcoming motivational difficulties associated with lifestyle interventions. An initial positive experience with weight loss may facilitate the implementation of the recommended lifestyle changes [22]. To ascertain the long-term outcomes of the study, participants will be followed up for a further 12 months after the end of the intervention. The results of this long-term follow-up will be reported in due course.

### Strenghts and limitations

The principal strength of this study is the inclusion of patients from daily real-world clinical practice, with straightforward and simple inclusion criteria, which enhances the generalisability of the findings. Although the inclusion criteria were relatively flexible, the dropout rate was satisfactory, with 73% of participants completing the trial. Furthermore, the use of oral semaglutide and metformin was demonstrated to be feasible, effective, and safe in this population of psychiatric patients. Although the superiority of semaglutide over metformin could not be demonstrated due to the limited sample size, it can be concluded that semaglutide was at least as effective as metformin. The adverse effects were relatively mild and transient, and a positive effect on psychiatric symptoms and self-perceived quality of life was demonstrated.

As this study employed a non-randomised open-label design, in which patients and treating physicians jointly selected the medication to be used, it is possible that this may have introduced a bias into the study results. A recent meta-analysis demonstrated that a substantial placebo effect is observed in weight-loss trials, with up to 11% of participants losing more than 5% of their body weight after 6 months while taking a placebo drug [48]. It is possible that the placebo effect may be enhanced in our study due to the shared decision-making process, whereby patients receive lifestyle advice and increased attention from their treating clinician, which may result in favourable behavioural changes [48]. On the other hand, this method of shared decision-making represents a standard practice in clinical settings, offering a valuable insight into real-world scenarios. Further studies, preferably RCTs, are required to confirm these results. Secondly, the sample size was relatively small, particularly in the semaglutide group. This was likely due to patients' reluctance to utilise the newer medication and the higher costs involved, which may have constituted a barrier for some. As this study was conducted as part of routine clinical practice, the standard medications prescribed were covered by the patients' health insurance. However, at the time the study was conducted, the use of semaglutide for weight loss was not covered by the Dutch health insurance system, in contrast to the use of metformin. Furthermore, the discrepancy in patient numbers between the two groups can be attributed, at least in part, to the relative novelty of semaglutide as a therapeutic agent. Patients frequently exhibited reluctance to utilise this medication, in part due to concerns about potential adverse effects that have recently received media attention. While weight loss was more pronounced with semaglutide, we were unable to demonstrate a significant difference with metformin. Thirdly, the dose of semaglutide employed in this study was 7 mg, which is considerably below the approved dose for the management of obesity. It is plausible that a higher dose may have resulted in greater weight loss, but probably also in a greater incidence of adverse effects. Additionally, the study did not include the combination of lifestyle interventions with either semaglutide or metformin. Consequently, it was not possible to demonstrate whether the combination of lifestyle changes and the addition of semaglutide or metformin in the treatment of AIWG leads to greater weight loss than lifestyle changes or the addition of semaglutide or metformin alone. Moreover, the study did not include data on withdrawal strategies for semaglutide and their effect on body weight. The results of the long-term follow-up may provide further insight into the question of whether patients regain weight after stopping the GLP-1 antagonist. Furthermore, the weight gain observed in the patient sample had already persisted for a period exceeding six months. It would be prudent to intervene at an earlier stage to prevent the occurrence of excessive weight gain. In addition, this study did not systematically control for the use of other psychotropic medication, including antidepressants and mood stabilisers, which are frequently used by this population. However, all psychotropic medications and doses remained unchanged throughout the study period, and no additional psychotropic medications were introduced during this period. It is therefore unlikely that the observed weight changes during the study period can be attributed to these medications. Additionally, there was no systematic measurement or control for tobacco use, despite evidence indicating that nicotine exerts an effect on GLP-1, which may contribute to reduced feelings of hunger [49]. Conversely, there is some evidence to suggest that GLP-1 agonists may assist in reducing or stopping smoking [50]. The ESM data from the present study may provide some insight into this phenomenon, although this hypothesis remains to be tested. As with psychotropic medication, the impact of tobacco consumption was controlled for by maintaining a consistent dose and drug regimen throughout the study period. Furthermore, the study duration was relatively short. Consequently, further studies with longer follow-up periods are required to confirm the long-term efficacy and safety of these treatments in this population. Finally, given the exploratory nature of our study and the small sample size, we did not control for multiple testing, which should be kept in mind when considering or interpreting the results.

## Conclusions

Oral semaglutide has been demonstrated to be a viable, effective, and safe treatment option for psychiatric patients. Although the study did not demonstrate that semaglutide is more efficacious than metformin, it can be concluded that semaglutide is at least as effective as metformin. Moreover, the adverse effects were generally mild and transient, and the treatment demonstrated a favourable impact on psychiatric symptoms and self-perceived quality of life.

## Data Availability

The datasets generated and analysed during the current study are not publicly available due to the restrictions imposed by ethical and data protection regulations. However, they are available from the corresponding author upon reasonable request.

## Abbreviations

AIWG: Antipsychotic-induced weight gain
AP(s): Antipsychotic(s)
BMI: Body mass index
BPRS: Brief Psychiatric Rating Scale
CI: Confidence interval
DM2: Diabetes mellitus type 2
ESM: Experience sampling method
GLP-1: Glucagon-like peptide-1
HbA1c: Glycated haemoglobin
HDL: High-density lipoprotein
IRB: Institutional review board
KQOL: Kemp Quality of Life scale
LDL: Low-density lipoprotein
MOSA: Management of Obesity with Semaglutide in AIWG
NMA: Network meta-analysis
QoL: Quality of life
SMI: Severe mental illness
UKU-SERS: UKU-Side Effect Rating Scale
